# Signaling pathways involved in vascular smooth muscle cell calcification during hyperphosphatemia

**DOI:** 10.1007/s00018-019-03054-z

**Published:** 2019-03-18

**Authors:** Jakob Voelkl, Florian Lang, Kai-Uwe Eckardt, Kerstin Amann, Makoto Kuro-o, Andreas Pasch, Burkert Pieske, Ioana Alesutan

**Affiliations:** 1 Institute for Physiology and Pathophysiology, Johannes Kepler University Linz, Altenberger Strasse 69, 4040 Linz, Austria; 2 Department of Internal Medicine and Cardiology, Charité–Universitätsmedizin Berlin, Campus Virchow-Klinikum, Augustenburgerplatz 1, 13353 Berlin, Germany; 3 DZHK (German Centre for Cardiovascular Research), Partner Site Berlin, 13347 Berlin, Germany; 4 Department of Nephrology and Medical Intensive Care, Charité–Universitätsmedizin Berlin, Augustenburgerplatz 1, 13353 Berlin, Germany; 5 Department of Physiology I, Eberhard-Karls University, Wilhelmstr. 56, 72076 Tübingen, Germany; 6 Department of Nephropathology, Universität Erlangen-Nürnberg, Krankenhausstr. 8-10, 91054 Erlangen, Germany; 7 Center for Molecular Medicine, Jichi Medical University, 3311-1 Yakushiji, Shimotsuke, Tochigi 329-0498 Japan; 8 Calciscon AG, Aarbergstrasse 5, 2560 Nidau-Biel, Switzerland; 9 Berlin Institute of Health (BIH), Anna-Louisa-Karsch Str. 2, 10178 Berlin, Germany; 10 Department of Internal Medicine and Cardiology, German Heart Center Berlin (DHZB), Augustenburger Platz 1, 13353 Berlin, Germany

**Keywords:** Osteogenic signaling, Vascular smooth muscle cells, Vascular calcification, Phosphate, CKD

## Abstract

Medial vascular calcification has emerged as a putative key factor contributing to the excessive cardiovascular mortality of patients with chronic kidney disease (CKD). Hyperphosphatemia is considered a decisive determinant of vascular calcification in CKD. A critical role in initiation and progression of vascular calcification during elevated phosphate conditions is attributed to vascular smooth muscle cells (VSMCs), which are able to change their phenotype into osteo-/chondroblasts-like cells. These transdifferentiated VSMCs actively promote calcification in the medial layer of the arteries by producing a local pro-calcifying environment as well as nidus sites for precipitation of calcium and phosphate and growth of calcium phosphate crystals. Elevated extracellular phosphate induces osteo-/chondrogenic transdifferentiation of VSMCs through complex intracellular signaling pathways, which are still incompletely understood. The present review addresses critical intracellular pathways controlling osteo-/chondrogenic transdifferentiation of VSMCs and, thus, vascular calcification during hyperphosphatemia. Elucidating these pathways holds a significant promise to open novel therapeutic opportunities counteracting the progression of vascular calcification in CKD.

## Introduction

Medial vascular calcification, often termed in short vascular calcification, represents the pathological deposition of calcium and phosphate in the medial layer of the arteries [1]. Vascular calcification is observed as a common complication in chronic kidney disease (CKD) [2, 3], diabetes mellitus [4], and aging [5]. It is also found in association with various pathological conditions including hypertension, atherosclerosis, osteoporosis, and rheumatoid arthritis [6, 7], and can be caused by rare monogenic disorders [8, 9].

The most extensive vascular calcification is observed in patients with CKD [2, 3]. In these patients, vascular calcification was suggested as a critical risk factor for cardiovascular events, and is associated with increased cardiovascular and all-cause morbidity and mortality [10, 11]. Vascular calcification has been considered decisive for the clinical course of the disease [2]. Nonetheless, the exact contribution of vascular calcification to cardiovascular mortality remains to be established, as currently only observational data are available. The observational studies are hampered by the slow onset and progression of vascular calcification and limited diagnostic methods. The recent discovery of a nanoparticle-based test of the calcification propensity may, however, establish a clinical approach to study risk factors and mechanisms of vascular calcification in CKD [12].

However, so far, the complex processes leading to vascular calcification in CKD remain incompletely understood. Consequently, no convincing concepts and treatment options to prevent or reduce the development of vascular calcification are yet available [13, 14].

## Phosphate and vascular calcification in CKD

In CKD, the initiation and progression of vascular calcification is triggered by a combination of various pathological factors [2, 3, 15, 16]. Dysregulation of mineral homeostasis and elevated phosphate levels are considered key determinants of vascular calcification in CKD [1, 17]. Hyperphosphatemia frequently occurs as a consequence of impaired renal function [2] and is associated with vascular calcification [2, 6] as well as increased risk for cardiovascular events and death [18]. However, the effects of phosphate binders on vascular calcification are still uncertain [19], which may be also attributed to the complex interplay of systemic phosphate handling and the availability of bone as a large source of phosphate [20]. Phosphate handling and development of hyperphosphatemia in CKD have been reviewed in detail elsewhere [20–24].

Phosphate complexes with calcium and calcium phosphate nanoparticles are able to activate pro-calcific intracellular signaling pathways [25, 26]. Increased calcium phosphate product levels [27] and calcium phosphate–protein complexes, known as calciprotein particles (CPPs) [28–30], are associated with the development of vascular calcification in CKD. The formation of CPPs and mineral stress have been reviewed in detail elsewhere [31, 32].

Even in patients with normal renal function, enhanced serum phosphate levels are associated with coronary artery calcification [33] and a high risk of cardiovascular events and mortality [34]. Thus, phosphate seems to play a crucial role in the pathophysiology of vascular calcification [1, 29].

## Mechanisms of vascular calcification in hyperphosphatemia

**Fig. 1:**
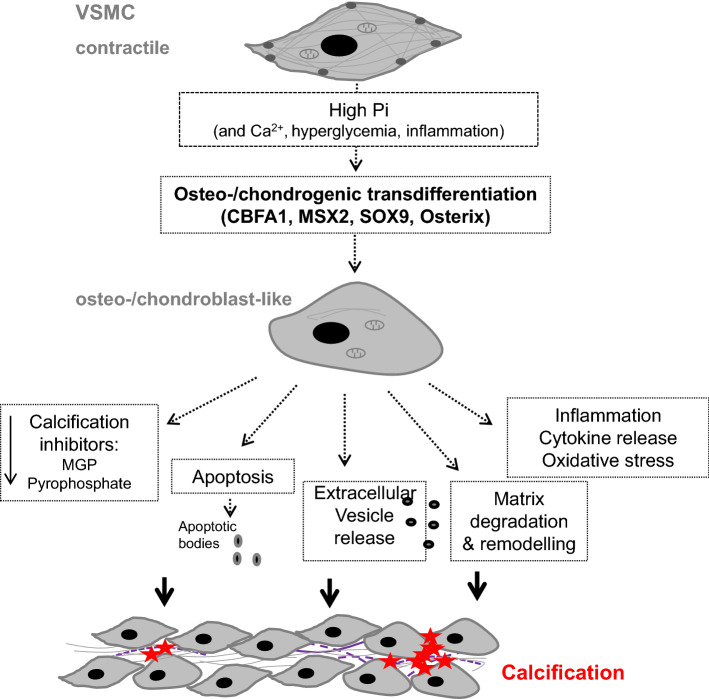
Role of vascular smooth muscle cells in vascular calcification. Following exposure to pro-calcific factors, most importantly hyperphosphatemia, vascular smooth muscle cells (VSMCs) are able to transdifferentiate into an osteo-/chondrogenic phenotype. This process is characterized and, at least partly, mediated by expression of osteogenic transcription factors such as CBFA1, MSX2, SOX9, and osterix. The osteo-/chondroblast-like cells actively promote calcification by reduced availability of calcification inhibitors, apoptosis, and apoptotic body release as well as release of calcifying extracellular vesicles, remodeling of the extracellular matrix and elastin degradation, and a pro-inflammatory state with release of pro-inflammatory cytokines and oxidative stress. These create a pro-calcifying environment, which allows for active mineralization of the vasculature

The mechanisms promoting the initiation and progression of vascular calcification show similarities to those accomplishing physiological bone formation [35, 36] involving osteo-/chondrogenic transdifferentiation as well as apoptosis of vascular cells, decreased availability of calcification inhibitors, extracellular vesicle release, and remodeling of extracellular matrix [2, 6]. These mechanisms are not mutually exclusive. Vascular smooth muscle cells (VSMCs) play a key role during vascular calcification (Fig. 1) [3, 6, 15, 17].

Under physiological conditions, calcium and phosphate concentrations exceed their solubility [37] and endogenous local and circulating calcification inhibitors are required to prevent the ectopic precipitation of calcium and phosphate [2]. The strongest endogenous inhibitor of mineralization is considered to be inorganic pyrophosphate [3, 6] found in relatively high levels in blood [1], but also being locally produced by VSMCs [10]. VSMCs produce and release pyrophosphate in the extracellular space, effects involving the ectonucleotide pyrophosphatase/phosphodiesterase (ENPP1) and the transmembrane protein ankylosis protein homolog (ANKH) [10, 38]. In addition, Fetuin-A is a circulating protein that can bind directly calcium or hydroxyapatite to inhibit the growth of hydroxyapatite crystals [1, 2]. Dietary protein restriction in rats with CKD is associated with reduced systemic Fetuin-A concentrations and increased vascular calcification [39]. VSMCs can take up Fetuin-A from the extracellular space and produce several other inhibitory proteins such as matrix-Gla protein, osteopontin, or osteoprotegerin [1, 2], which are further loaded into extracellular vesicles to prevent vascular mineralization [3]. Various pathological factors, especially high extracellular phosphate levels, are able to suppress the production of calcification inhibitors and to promote the release of exosomal vesicles lacking these inhibitors, but with increased load of pro-calcific proteins such as tissue-nonspecific alkaline phosphatase (ALPL) [40, 41]. These could form microcalcifications and serve as a nidus for calcium phosphate precipitation and growth of calcium phosphate crystals [7, 40, 42].

Hyperphosphatemia may further lead to extracellular matrix remodeling in the medial layer of the vasculature [2, 43]. Increased production of matrix metalloproteinases (MMPs) by VSMCs [44–47] such as MMP2 or MMP9 [43, 46, 47] and further degradation of various extracellular matrix proteins including elastic fibers provide additional nidus sites for calcium phosphate precipitation [2, 7, 43]. Along those lines, excessive levels of the cysteine protease cathepsin S leads to cleavage of elastin and generation bioactive elastin peptides [48], which may act directly on VSMCs to further accelerate vascular calcification [2, 48, 49] during hyperphosphatemia. Furthermore, in the presence of phosphate, VSMCs synthesize increased levels of collagen, leading to deposition of a collagen-enriched extracellular matrix [7, 10, 50, 51]. More importantly, phosphate induces the expression of enzymes such as procollagen-lysine, 2-oxoglutarate 5-dioxygenase 1 (PLOD1), or lysyl oxidase (LOX) in VSMCs [52] that mediate collagen cross-linking and supramolecular organization [53], which seems to represent a critical event during extracellular matrix remodeling associated with vascular tissue mineralization [50, 52].

Apoptosis is another key mechanism promoting vascular tissue mineralization [54, 55]. Previous studies showed that high extracellular phosphate levels induce apoptosis and necrosis of VSMCs [1, 2, 16, 46]. Under such circumstances, VSMCs release apoptotic bodies, which could serve as a nidus for calcium phosphate deposition [1, 42, 54, 56]. In addition, apoptosis of VSMCs may lead to medial VSMC loss and degeneration as well as elastin breaks, cyst-like structures, and changes in extracellular matrix composition within the medial layer of the arteries [55, 57], effects that may also contribute to vascular mineralization [2, 7, 43]. Inhibition of apoptosis with caspase inhibitor is able to reduce VSMC mineralization [54].

In vascular tissue, calcification is actively promoted by osteoblast- and chondroblast-like cells [36, 58]. In the media, VSMCs are able to change their phenotype from a contractile into an osteo-/chondrogenic phenotype under high phosphate conditions [6, 17]. Osteo-/chondrogenic transdifferentiation of VSMCs precedes and is required for vascular tissue mineralization [17, 58, 59]. Further sources of osteo-/chondroblast-like cells in the vasculature include pericytes [60], myofibroblasts in the adventitia [7, 61], or vascular progenitor cells [62].

### Phosphate-induced osteo-/chondrogenic transdifferentiation of VSMCs

In response to high extracellular phosphate levels, VSMCs are able to change their phenotype into osteo-/chondroblast-like cells actively promoting vascular mineralization [46, 63, 64]. These transdifferentiated VSMCs lose their contractile phenotype in favor of a more mesenchymal one and gain similar properties as osteoblasts and chondroblasts [7]. They express osteogenic transcription factors such as msh homeobox 2 (MSX2), core-binding factor α-1 (CBFA1) [46, 63–65] or osterix [66], as well as chondrogenic transcription factors including SRY-Box 9 (SOX9) [46, 67–69]. The transcription factor CBFA1 (also known as RUNX2) plays a decisive role in vascular calcification [3]. Deficiency of CBFA1 in VSMCs prevents vascular osteo-/chondrogenic transdifferentiation and calcification [70, 71]. The transcription factor MSX2 induces the expression of CBFA1 and osterix in VSMCs [72]. Osterix is up-regulated by CBFA1 [73] and required for its full activation [10]. In addition, SOX9 may cooperate with CBFA1 to suppress the smooth muscle phenotype and promote transdifferentiation of VSMCs [69].

In these VSMCs, the expression of smooth muscle-specific proteins such as α-smooth muscle actin (αSMA) or smooth muscle protein 22-α (SM22-α) is reduced [58]. The phosphate-induced increase of osteogenic transcription factor expression is considered an event prior to downregulation of VSMC-specific markers [74, 75].

The osteo-/chondrogenic transcription factors further induce the expression of osteogenic- and chondrogenic-specific proteins in VSMCs such as osteocalcin, type I collagen, bone morphogenetic protein-2 (BMP-2), or ALPL [2, 6, 63, 76, 77]. ALPL is a key regulator of vascular calcification [7, 76]. Increased ALPL activity is a decisive event in vascular calcification [7]. Similar as in bone, ALPL degrades inorganic pyrophosphate to allow unrestrained tissue mineralization [7, 10].

The osteo-/chondrogenic transdifferentiated VSMCs may promote the calcification of vascular tissue by producing a local pro-calcifying environment and nidus sites for precipitation of calcium and phosphate as well as growth of calcium phosphate crystals.

## Signaling pathways regulating VSMCs calcification during high phosphate conditions

Extracellular phosphate is a signaling molecule [78] that induces various changes in VSMCs via the regulation of intracellular pathways [46, 51, 63, 64]. The signaling pathways controlling osteo-/chondrogenic transdifferentiation of VSMCs and, thus, vascular calcification under elevated phosphate conditions are extremely complex and still incompletely understood. Identification of the critical intracellular pathways regulating phosphate-induced vascular calcification may provide the basis for therapeutic strategies to reduce the progression of vascular calcification in CKD. A significant progress has been made in this field and some recent observations are highlighted in this review.

### Transduction of phosphate signals to VSMCs

How VSMCs sense elevated extracellular phosphate levels is still ill defined. Toll-like receptors may be involved in phosphate-sensing [79]. In addition, calcium phosphate nanoparticles can be internalized and dissolved in lysosomes, thus, triggering intracellular signaling effects [6]. Phosphate can be transported into VSMCs via the type III sodium-dependent phosphate transporters PIT1 and PIT2 [2, 15].

PIT1 is well described to mediate the effects of phosphate in VSMCs via phosphate transport-dependent and phosphate transport-independent functions [80, 81]. Interestingly, PIT1 is most abundant in cells at the endoplasmic reticulum [82] and the exact mechanisms how PIT1 mediates its effects on vascular calcification are not finally defined. PIT1-downstream signaling involves the activation of ERK1/2 MAP-kinase [75, 81] and leads to up-regulation of CBFA1 and ALPL expression in VSMCs [15, 80, 81]. The downstream effects of ERK1/2 during vascular calcification are still incompletely understood. Silencing of PIT1 is sufficient to suppress phosphate-induced osteoinduction and mineralization of VSMCs [80]. PIT1 functions are apparently modulated mainly by changes in expression levels [66, 83]. Accordingly, phosphate up-regulates PIT1 expression in VSMCs [64, 84]. PIT1 expression can be transcriptionally regulated in VSMCs by several pathways including SAPK/JNK MAP-kinase signaling [85], β-catenin signaling [84], or transcription factor-4 (ATF4) [86]. Activation of the mineralocorticoid receptor (MR) in VSMCs may directly up-regulate PIT1 transcription [59, 66]. PIT1 is involved in the pro-calcific effects of aldosterone in VSMCs [66]. Elevated phosphate levels induce the expression of aldosterone synthase in VSMCs via disruption of apurinic/apyrimidinic endodeoxyribonuclease 1 (APEX1)-dependent gene suppression, while MR blockade is able to reduce PIT1-dependent osteoinductive signaling and calcification of VSMCs during hyperphosphatemia [64].

PIT2 is up-regulated together with PIT1 in the vasculature in uremic conditions [87]. These two transporters were considered to play a redundant role in phosphate-induced osteoinduction in VSMCs [88]. However, the recent findings suggest that PIT2 may even protect against vascular calcification by the up-regulation of osteoprotegerin [89], a key regulator of bone metabolism and inhibitor of vascular calcification [31, 90].

### Phosphate-dependent osteoinductive signaling cascades

Presumably, a multitude of intracellular signaling pathways and para/autocrine signals orchestrate the calcification response to phosphate. Elevated extracellular phosphate levels trigger activation of the transcription factor nuclear factor “‘kappa-light-chain-enhancer” of activated B cells (NF-kB) in VSMCs [63, 79, 91, 92]. Activation of the NF-kB pathway is critically important for phosphate-induced vascular calcification [63, 79, 91–93]. NF-kB signaling promotes VSMC mineralization in part by inducing MSX2 expression and up-regulating CBFA1 to increase ALPL expression [63, 72, 93]. Moreover, NF-kB increases the expression of tristetraprolin (TTP), an RNA-destabilizing protein that reduces *ANKH* mRNA levels and, thus, may modify the production or release of pyrophosphate in the extracellular space [91, 93]. In addition, smooth muscle-specific deficiency of NF-KB or NF-kB inhibition is sufficient to block vascular calcification during hyperphosphatemia [63, 79, 91, 92].

The deleterious effects of phosphate in the cardiovascular system may also involve the serum- and glucocorticoid-inducible kinase (SGK1) [94]. SGK1, a serine/threonine protein kinase, is activated via phosphatidylinositide-3-kinase (PI3K), 3-phosphoinositide-dependent kinase 1 (PDK1), and mammalian target of rapamycin (mTOR) signaling [95], and orchestrates the cellular response to various pathological triggers [94–98]. In VSMCs, SGK1 expression and activity are increased by phosphate and its activation plays a key role in phosphate-induced vascular calcification [93]. Inhibition or deficiency of SGK1 is able to suppress vascular calcification during hyperphosphatemia [93]. SGK1 promotes osteo-/chondrogenic transdifferentiation and calcification of VSMCs through the activation of the transcription factor NF-kB [93]. SGK1 directly phosphorylates IKKα to induce NF-kB activation, an effect requiring also IKKβ [99]. Furthermore, phosphorylation-dependent ubiquitination and degradation of IkBα leads to NF-kB nuclear translocation, NF-kB-dependent target gene transcription [63, 94], and subsequent osteo-/chondrogenic transdifferentiation of VSMCs. Thus, interference with SGK1/NF-kB signaling pathway may preserve an anti-calcific environment of VSMCs and ameliorate vascular calcification during hyperphosphatemia.

AKT (also known as protein kinase B) signaling contributes to the complex machinery underlying VSMC osteoinduction [56, 100, 101]. Phosphate reduces AKT phosphorylation in VSMCs [56, 100], while both pro-calcific effects [101] and protective effects against vascular calcification [101, 102] of AKT activation have been described. AKT and SGK1 are able to phosphorylate and inactivate glycogen synthase kinase 3 (GSK-3) [103, 104]. Vascular AKT/SGK-resistance of GSK-3 is able to ameliorate vascular osteoinduction and calcification [104]. The PI3K-dependent pathways, thus, have an essential, but diverse and complex role during vascular calcification, which requires further study to dissect pro- and anti-calcific effects.

The WNT/β-catenin pathway is established as a major component of the osteoinductive signaling cascade and a mediator of vascular calcification [84, 105–110]. WNT are ligand proteins that bind at the cell surface receptors of the Frizzled family and lipoprotein receptor-related protein (LRP)-5/6 for intracellular signaling [109], leading to β-catenin activation, nuclear translocation, and target gene expression [84, 109]. GSK3β activity promotes β-catenin phosphorylation and its degradation by the proteasome, interfering with WNT-signaling [103, 104]. WNT/β-catenin pathway is activated by phosphate [108, 110], and participates in phosphate-induced osteo-/chondrogenic transdifferentiation and calcification of VSMCs [84, 105, 106]. Activation of the WNT/β-catenin pathway is required for the downstream effects of MSX2 [105] and contributes to osteo-/chondrogenic transdifferentiation of VSMCs, at least in part, by directly up-regulating CBFA1 [106] as well as PIT1 gene expression [84] during hyperphosphatemia. In addition, WNT/β-catenin may further participate to vascular calcification by induction of MMP2 and MMP9 in VSMCs [47]. Furthermore, interference with WNT/β-catenin signaling activation is able to suppress osteo-/chondrogenic transdifferentiation of VSMCs and vascular calcification [107, 108, 110–112]. WNT/β-catenin pathway inhibitors such as Dickkopf-related protein 1 (DKK1) [108] or secreted frizzled-related proteins (SFRPs) [112] showed anti-calcific effects in VSMCs during in vitro hyperphosphatemia. However, the systemic effects of WNT/β-catenin may be more complex, as DKK1 inhibition also promoted bone formation and prevented vascular calcification in a CKD mouse model [113].

### Counterregulatory pathways during phosphate-induced VSMCs calcification

Phosphate-induced activation of NF-kB signaling in VSMCs can be counteracted by the endogenous inhibitor TNFAIP3, also known as A20 [63]. TNFAIP3 is a zinc-finger protein, which interferes with NF-kB activation via IkBα [114]. In accordance, up-regulation of TNFAIP3 expression is able to inhibit osteo-/chondrogenic transdifferentiation and calcification of VSMCs following phosphate exposure [63]. In VSMCs, TNFAIP3 expression can be increased at the transcriptional level by the elevated extracellular Zn^2+^ levels via the Zn^2+^-sensing receptor (ZnR), also known as G protein-coupled receptor 39 (GPR39) [63]. Both in vitro and in vivo Zn^2+^ supplementation up-regulates vascular TNFAIP3 expression and suppresses NF-kB-dependent osteo-/chondrogenic signaling as well as calcification of VSMCs during hyperphosphatemia [63].

Similarly, activation of another membrane receptor, the Ca^2+^-sensing receptor (CASR), may interfere with phosphate-induced VSMC calcification [51, 115, 116]. CASR can be activated by extracellular Ca^2+^, but also by the other cations such as Mg^2+^, Gd^3+^, amino acids, or polyamines [51, 117]. The downstream signaling involved in the anti-calcific effects of CASR activation is, however, still incompletely understood [51, 116]. Nonetheless, calcimimetics increase CASR expression and reduce mineralization of VSMCs [118]. Moreover, activation of the vitamin D_3_ receptor (VDR) inhibits VSMCs mineralization by up-regulating CASR expression [115]. Along those lines, Mg^2+^ supplementation showed inhibitory effects on vascular calcification in vitro and in animal models [119–121]. In addition to activation of the CASR [51], Mg^2+^ may also inhibit WNT/β-catenin signaling [110] or directly interfere with calcium phosphate precipitation [122] to suppress vascular tissue mineralization. The so far known mechanisms involved in the anti-calcific properties of Mg^2+^ are discussed in detail elsewhere [119–121]. Taken together, activation of ZnR-dependent as well as CASR-dependent anti-calcific intracellular pathways interferes with phosphate-induced signaling, osteo-/chondrogenic transdifferentiation of VSMCs, and, thus, vascular calcification.

Another factor that may interfere with osteo-/chondrogenic pathways in VSMCs is the FGF23 co-receptor α-klotho, which may also circulate as soluble humoral factor [123–125]. In VSMCs, according to some studies, klotho expression is down-regulated by phosphate [124], an effect associated with activated mTOR signaling [123], which augments vascular calcification [123, 124]. However, other studies found no evidence of klotho expression in VSMCs [126]. Soluble klotho has been suggested to contribute to vascular calcification [125] and may be able to inhibit the phosphate uptake via PIT1 in VSMCs [125]. The suggested protective effects of klotho on vascular calcification involve the inhibition of the WNT/β-catenin signaling pathway [127, 128]. In addition, both anti- [129] and pro-calcific [130] effects of FGF23 were described. Further research is required to elucidate the various functions and possible modifiers of the effects of vascular FGF23/klotho.

### Cytokine signaling and inflammatory responses

Pro-inflammatory intracellular signaling in VSMCs also seems to induce or augment osteo-/chondrogenic transdifferentiation of VSMCs triggered by elevated phosphate levels [13, 67, 68, 131–133]. Phosphate is associated with vascular inflammation [67, 68, 132, 134–136]. Accordingly, a recent study showed that phosphate overload directly induces local inflammation in cultured VSMCs and systemic and vascular inflammation in vivo [135]. VSMCs produce pro-inflammatory cytokines such as TNFα, IL-1β, IL-6, BMP-2, or TGFβ1 [66–68, 72, 137, 138], powerful stimulators of VSMC osteoinduction by modulating intracellular signaling [72, 138].

Exposure to calcium phosphate crystals induces IL-1β release via the activation of spleen tyrosine kinase (SYK), apparently independent from inflammasome activation [25]. Nonetheless, inflammasome activation is required for vascular calcification during hyperphosphatemia [137], effects presumably involving TNFα [139]. TNFα further increases MSX2 expression in VSMCs via the NF-kB pathway to induce osteo-/chondrogenic transdifferentiation of VSMCs [72]. In addition, the RANKL/RANK system augments vascular calcification via NF-kB, which can be blocked by the inhibitory RANKL-decoy receptor osteoprotegerin [140]. Similarly, the SGK1/NF-kB osteoinductive pathway may be activated by IL-18 [133], resulting in aggravation of phosphate-induced VSMCs mineralization [133, 141]. Furthermore, together with the NF-kB pathway, WNT/β-catenin signaling may modulate pro-inflammatory signaling cascades in VSMCs in response to hyperphosphatemia [127, 142, 143].

In addition, TNFα-mediated VSMC calcification is also associated with increase of BMP-2 signaling [144]. Phosphate induces BMP-2 expression in VSMCs [26, 79] and BMP-2 mediates the effects of phosphate in vascular calcification [134, 145]. BMP-2 triggers VSMC osteoinduction [146], at least partly, via MSX2 up-regulation [147] and involves the WNT/β-catenin pathway [145] as well as generation of cellular oxidative stress [148]. Furthermore, the pro-calcific effects of BMP-2 in VSMCs involve the up-regulation of PIT-1 expression [134] and SMAD signaling [148].

IL-6 is another key mediator of phosphate-induced vascular calcification [135, 149–151]. In VSMCs, IL-6 regulates various pathways leading to osteo-/chondrogenic transdifferentiation of VSMCs including activation of BMP-2-WNT/β-catenin signaling [152], RANKL [149], and STAT3 pathway [153, 154] or induction of oxidative stress [138, 151].

Moreover, TGFβ1 was described as a strong promoter of osteoinduction and calcification of VSMCs [67, 68, 155]. TGFβ1 expression is increased by phosphate in VSMCs [67, 68]. TGFβ1-downstream osteoinductive signaling includes the transcription factor NFAT5 (also known as TonEBP), which mediates the SOX9-dependent up-regulation of CBFA1 in VSMCs [67, 68, 155, 156]. TGFβ1 may also contribute to vascular calcification by inducing cellular senescence, including up-regulation of plasminogen activator inhibitor PAI-1, which exerts pro-calcific effects [67, 155, 157]. Most importantly, inhibition of TGFβ1-dependent signaling is able to suppress phosphate-induced vascular calcification [67, 68, 155].

### Signaling pathways induced by calcium phosphate nanoparticles and CPPs

Phosphate affects VSMCs calcification through intracellular effects of calcium phosphate nanoparticles [26], which can be engulfed by lysosomes [6, 63]. Calcium phosphate nanoparticles are endocytosed by VSMCs, which further leads to release of Ca^2+^ from the lysosomes, elevated intracellular Ca^2+^ levels, and subsequent apoptosis of VSMCs [6, 136] or Ca^2+^-induced inflammasome activation [158]. The osteogenic lysosomal effects are dependent on the acidic lysosomal pH [6, 136]. Accordingly, alkalinisation of lysosomal pH with NH_4_Cl [67], bafilomycin A1, or methylamine [68] is able to suppress phosphate-induced VSMCs osteo-/chondrogenic transdifferentiation and calcification. Lysosomes may also regulate several other osteoinductive signaling pathways, besides inducing apoptosis of VSMCs. The transfer of lysosomes loaded with LDL/cholesterol from macrophages into VSMCs may trigger the phenotypical transdifferentiation of VSMCs [159]. Moreover, lysosomes are involved in maturation of various proteins, including pro-TGFβ [160], which may affect VSMCs osteo-/chondrogenic transdifferentiation [67, 68, 119]. Thus, the lysosomes are key organelles for the intracellular osteoinductive effects of phosphate [6, 67, 68, 136].

Calcium phosphate crystal formation is inhibited by Fetuin-A and the formation of calciprotein particles (CPPs) [31]. However, these may undergo transition from the amorphous (primary CPPs) to the crystalline (secondary CPPs) phase [12, 30, 161], which is promoted by various physico-chemical factors [30]. The secondary CPPs may also trigger vascular calcification via a cell-mediated process [28, 161, 162] by inducing osteo-/chondrogenic transdifferentiation of VSMCs [28]. Secondary CPPs, but not primary CPPs, are able to trigger directly VSMC calcification [161, 162]. These effects involve the uptake of CPPs by VSMCs and an increase of intracellular Ca^2+^ levels [161] followed by the induction of cellular oxidative stress and pro-inflammatory responses [161, 162] to promote mineralization. Thus, due to its antioxidant properties, hydrogen sulfide suppresses VSMC calcification promoted by CPPs [162]. Increased expression and release of the pro-inflammatory cytokine TNFα and activation of TNFα/TNFR1 system is critically important for CPPs-induced VSMC calcification [161].

The properties of CPPs formation may also be utilized for novel diagnostic approaches [12]. A nanoparticle-based assay was developed, which detects the influence of serum on spontaneous transformation of primary CPPs into secondary CPPs and, thus, the balance between inhibitors and promoters of calcification in the serum [12]. Serum calcification propensity was suggested as a biomarker for cardiovascular disease [163] and shown to predict cardiovascular and all-cause mortality in CKD [164, 165].

### Oxidative stress-downstream signaling pathways

Oxidative stress contributes to vascular calcification in CKD [166]. Phosphate induces oxidative stress in VSMCs by triggering an imbalance between the antioxidant and the reactive oxygen species (ROS)-generating systems [10, 46, 138, 167]. Similarly, the superoxide-generating NAPDH oxidase system is associated with vascular calcification [168]. Oxidative stress mediates, at least partly, the effects of phosphate on osteo-/chondrogenic transdifferentiation and calcification of VSMCs [46, 138, 169]. In contrast to the acidification of lysosomes, intracellular alkalinisation by phosphate uptake via PIT1 [170] may contribute to phosphate-induced ROS production and oxidative stress [46, 170].

The downstream effectors of oxidative stress leading to osteo-/chondrogenic transdifferentiation of VSMCs include a multitude of signaling pathways. Oxidative stress is a strong promoter of CBFA1 expression and osteo-/chondrogenic transdifferentiation of VSMCs [169, 171, 172]. Furthermore, oxidative stress promotes osteoinduction in VSMCs via the ERK1/2 MAP-kinase [171, 172] as well as p38 MAP-kinase pathways [171]. Activation of either ERK1/2 [81, 171, 172] or p38 MAP-kinase signaling pathways [171, 173] promotes vascular calcification. P38 MAP-kinase may directly activate CBFA1 [173], contribute to inflammasome activation [174], and activate NF-kB via mitogen- and stress-activated protein kinase-1 (MSK1) [175]. ROS were also shown to activate the transcription factor NF-kB and NF-kB-dependent osteoinductive signaling pathways [167] and promote pro-inflammatory responses of VSMCs [176]. Oxidative stress leads to the increased expression of matrix metalloproteinases [46, 177] that degrade extracellular matrix to allow mineralization [43, 44] as well as increased PAI-1 expression [16, 46, 155]. In addition, oxidative stress may induce the apoptosis of VSMCs [178] to promote vascular mineralization [1].

In accordance, antioxidants blunt osteo-/chondrogenic transdifferentiation of VSMCs and vascular calcification [179]. Similarly, Fibulin-3, an extracellular matrix glycoprotein, inhibits phosphate-induced phenotypical transdifferentiation and calcification of VSMCs through inhibition of cellular oxidative stress [46]. Conversely, loss of cytosolic serine hydroxymethyl transferase 1 (SHMT1), a key enzyme in one carbon metabolism, aggravates VSMC osteoinduction and calcification during elevated phosphate conditions by inducing oxidative stress [180]. SHMT1 is, however, up-regulated following phosphate treatment in VSMCs, a finding pointing to a role of SHMT1 in the cellular response that limits calcification [180].

Nitric oxide (NO) levels are associated with oxidative stress in VSMCs [16]. NO deficiency may induce oxidative stress [16, 181] and, thus, may promote osteo-/chondrogenic transdifferentiation of VSMCs with subsequent mineralization of vascular tissue [16]. Impaired NO production leads to the aggravation of phosphate-induced vascular calcification [16]. Conversely, NO prevents vascular calcification and inhibits osteo-/chondrogenic signaling pathways by interfering with TGFβ1/PAI-1 signaling [16, 155]. Thus, NO is a key factor that regulates intracellular signaling pathways controlling vascular calcification.

### Apoptosis signaling pathways

In response to phosphate, a multitude of up-stream signaling cascades may lead eventually also to the activation of pro-apoptotic signaling pathways in VSMCs [6, 56, 74, 178, 182, 183]. A key event is represented by the downregulation of growth arrest-specific gene 6 (Gas6) and its receptor tyrosine kinase Axl [74, 182, 183]. Phosphate reduces the expression of Gas6 and Axl in VSMCs [184], leading to Bcl2 inactivation, activation of the pro-apoptotic protein Bcl2-associated death promoter (Bad), and subsequent caspase-3 activation and VSMC apoptosis [74, 182]. Gas6/Axl activates Bcl2 via AKT [183], a key downstream signaling pathway of the Gas6-mediated VSMC survival [183]. In accordance, phosphate inhibits AKT phosphorylation in VSMCs [56, 100, 183], while activation of the PI3K/AKT pathway may prevent phosphate-induced apoptosis of VSMCs [56]. Similarly, vitamin K2 [182], iron citrate [185], estrogens [186], testosterone [187], α-lipoic acid [188], or statins [183, 184] inhibit phosphate-induced VSMCs apoptosis by restoring the Gas6-dependent anti-apoptotic pathway, effects leading to a reduction in VSMC calcification [54, 55]. A key up-stream regulator of Gas6 expression in VSMCs is the AMP-activated protein kinase (AMPK) [189, 190]. AMPK activity is suppressed in the presence of phosphate [191] and AMPK activation reduces VSMC calcification [189–191], effects involving inhibition of oxidative stress-mediated apoptosis [189].

### Other factors involved in the regulation of osteoinductive pathways

Several additional cellular factors such as components of the epigenetic regulation [192] including microRNAs [193], DNA methylation [194–196], or histone modifications [197] contribute to the osteoinductive intracellular signaling pathways during hyperphosphatemia. The role of epigenetics [192] as well as the so far known microRNAs [193, 198, 199] involved in vascular calcification have been reviewed in detail elsewhere. The microRNAs were shown to have a decisive role in osteo-/chondrogenic transdifferentiation of VSMCs by regulating various cellular processes during hyperphosphatemia such as gene expression [192, 193, 198–202], inflammasome activation [137], apoptosis [201, 203], senescence [154, 201], or endoplasmic reticulum stress [203]. Moreover, the recent findings describe that microRNAs are involved in the regulation of several intracellular pathways controlling osteo-/chondrogenic phenotypic switch of VSMCs including the WNT/β-catenin pathway [107], PI3K signaling [204], STAT3 pathway [154], or TGFβ1/SMAD signaling [205].

In addition, aging-related epigenetic changes were shown to influence vascular calcification [198, 201, 206–209]. Hyperphosphatemia promotes premature senescence and aging of VSMCs [206, 210], at least in part, by suppressing Sirtuin 1 expression [201, 211] and subsequent p21 activation [211]. During senescence, VSMCs are characterized by telomere shortening, increased oxidative DNA damage or impaired DNA repair [212], factors driving osteo-/chondrogenic transdifferentiation of VSMCs [207–209]. Furthermore, senescent VSMCs are associated with increased expression of BMP-2 as well as pro-inflammatory cytokines such as IL-1β, IL-6, or TNFα [209], well-known promoters of VSMC calcification [72, 146, 147, 151]. In accordance, preventing cellular senescence by increasing Sirtuin 1 levels in VSMCs is able to interfere with phosphate-induced VSMC osteo-/chondrogenic transdifferentiation and calcification [211, 213].

Other cellular factors, which contribute to the osteoinductive intracellular pathways during hyperphosphatemia, include autophagy [214, 215], endoplasmic reticulum stress [172, 203], or mitochondrial dysfunction [188, 189, 213]. Increase of cellular autophagy [185, 214, 216], restoration of mitochondrial function [188, 189, 213], or inhibition of endoplasmic reticulum stress [172] were all described to interfere with phosphate-induced vascular calcification.

### Interplay between signaling pathways in the regulation of vascular calcification

**Fig. 2:**
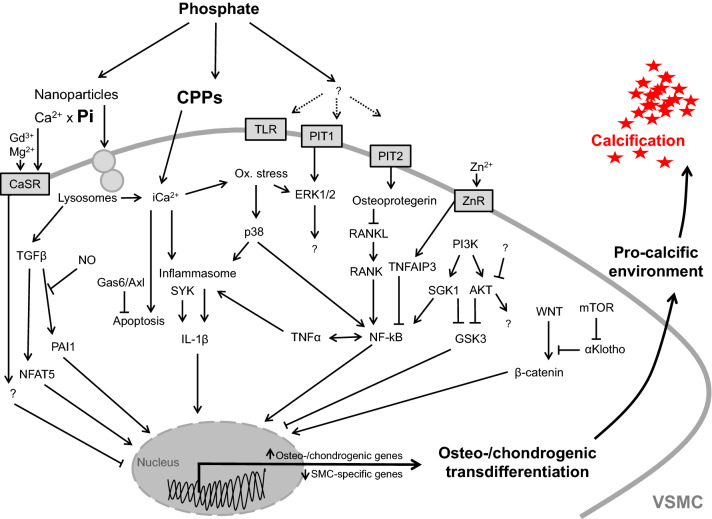
Critical signaling pathways involved in osteo-/chondrogenic transdifferentiation of vascular smooth muscle cells. Simplified schematic illustration of important phosphate-induced signaling pathways discussed in this review, ultimately leading to osteo-chondrogenic transdifferentiation of vascular smooth muscle cells (VSMCs), development of a pro-calcifying environment, and vascular calcification. For details and abbreviations, see the full text

Phosphate triggers osteo-/chondrogenic transdifferentiation and calcification of VSMCs by regulating a multitude of signaling pathways. As described above, these processes are controlled by an extremely complex cellular network of signaling pathways characterized by many cross talks and close interactions between these signaling cascades (Fig. 2). Alteration of central factors of this network affects also on the interconnected signaling pathways and, thus, may be effective in interfering with the pro-calcific effects of phosphate in VSMCs.

Clearly, elucidating the complex interplay of the cellular responses to high phosphate exposure requires further study, to connect the currently identified pathways, to define the relative importance during different stages of the process, and, finally, to gain a more comprehensive understanding of the onset and progression of vascular calcification in CKD.

## Conclusions

Elevated phosphate concentrations trigger vascular calcification through a complex and highly regulated process. A key role during vascular calcification is attributed to VSMCs, which actively promote mineralization by mechanisms involving osteo-/chondrogenic phenotypical transdifferentiation. A complex interplay between different intracellular signaling cascades tightly controls phosphate-induced osteo-/chondrogenic transdifferentiation of VSMCs. Identification of the critical intracellular pathways regulating vascular calcification may help to develop feasible therapeutic approaches to reduce the progression of vascular calcification in CKD.
